# Recent Insights Into Processing Approaches and Potential Health Benefits of Goat Milk and Its Products: A Review

**DOI:** 10.3389/fnut.2021.789117

**Published:** 2021-12-06

**Authors:** Gulzar Ahmad Nayik, Yash D. Jagdale, Sailee A. Gaikwad, Anupama N. Devkatte, Aamir Hussain Dar, Daniel Severus Dezmirean, Otilia Bobis, Muhammad Modassar A. N. Ranjha, Mohammad Javed Ansari, Hassan A. Hemeg, Saqer S. Alotaibi

**Affiliations:** ^1^Department of Food Science & Technology, Government Degree College Shopian, Jammu and Kashmir, India; ^2^Maharashtra Institute of Technology (MIT) School of Food Technology, Art, Design and Technology University, Pune, India; ^3^Department of Food Technology, Islamic University of Science & Technology, Awantipora, India; ^4^Department of Technological Sciences, Faculty of Animal Science and Biotechnology, University of Agricultural Sciences and Veterinary Medicine Cluj-Napoca, Cluj-Napoca, Romania; ^5^Institute of Food Science and Nutrition, University of Sargodha, Sargodha, Pakistan; ^6^Department of Botany, Hindu College Moradabad (Mahatma Jyotiba Phule Rohilkhand University Bareilly), Moradabad, India; ^7^Department of Medical Laboratory Technology, College of Applied Medical Sciences, Taibah University, Medina, Saudi Arabia; ^8^Department of Biotechnology, College of Science, Taif University, Ta'if, Saudi Arabia

**Keywords:** goat milk, pasturized milk, health benefits, processing, milk products

## Abstract

Goat milk is considered to be a potential source of various macro- and micro-nutrients. It contains a good proportion of protein, fat, carbohydrates, and other nutritional components which help in promoting nutritional and desirable health benefits. Goat milk is considered to be superior in terms of numerous health benefits, and lower risk of allergy, when compared to the milk of other species. Several processing techniques such as pasteurization, ultrafiltration, microfiltration, and ultrasound have been employed to enhance the quality and shelf life of goat milk and its products. The diverse range of goat milk-based products such as yogurt, cheese, fermented milk, goat milk powder, and others are available in the market and are prepared by the intervention of advanced processing technologies. Goats raised in pasture-based feeding systems are shown to have a better milk nutritional composition than its counterpart. Goat milk contains potential bioactive components, which aids in the maintenance of the proper metabolism and functioning of the human body. This review gives insight into the key nutritional ingredients and bioactive constituents present in goat milk and their potential role in the development of various functional foods using different processing technologies. Goat milk could be considered as a significant option for milk consumption in infants, as compared to other milk available.

## Introduction

The goathas been known as the oldest domesticated animal in mankind's history as a source of milk and different dairy products, required for survival as well as fulfilling the need for a nutritious and balanced diet. Although goats only contribute around 2% production supply of the world's total milk, their significance in the economic upliftment and nutritional well-being of the human populationis crucial in several regions around the world, particularly in the Middle East and Mediterranean countries (1). Goats have been categorized into a variety of breeds and are raised in different environments across the world. The goat breeds specifically meant for milk production and dairy products are receiving more focus on a study regarding milk quality and its yield (2). Many recent international and national conferences proceedings have highlighted and demonstrated the relevance of goats as global producers of vital food in the form of meat and dairy products (3). There are ~1 billion goats on the planet, and the global goat population has doubled over the last four decades. According to the Food and Agriculture Organization (FAO), developing countries are home to more than 90% of the world's goat population; Asia has the largest proportion of the world's goat population, followed by Africa (4). Goat is considered to be valuable to local farmers for both economic and subsistence reasons, by contributing to the enhancement of the livelihood of marginal farmers (5). The global output of goat milk is projected to be ~15,500,000 tons per year, with the developing nations accounting for 83 percent of the total (6).

Currently, non-bovine annual milk production stands at 133 million tons, representing more than 17% of total milk output worldwide. Goat milk production represents 13.5% of the total output of non-bovine milk, making it the most significant contributor (7). Goat milk is generally consumed directly or used to make a diverse range of dairy products, and it is estimated that a large population consumes goat milk and its products across the world (8, 9). Because a large portion of domestic non-commercial consumption and production remains unrecorded, the goat milk yield is likely to be considerably greater than what these official figures suggest (10). Due to the natural reproductive cycles, the availability of food during the rainy season, and leftover feed from seasonal harvest, there is yearly fluctuation in goat milk, production and is seasonal (11). Consumption of goat milk like that of other ruminant species is impacted by different factors involving individual features, breed, handling, stage of lactation, the composition of feed, climate, and vast differences not only across but also within the system of production (12, 13). Studies concluded that it is possible to modify the fatty acid composition of ruminant products by manipulating their diet with appropriate dietary fat sources. An interesting characteristic of goats is the fact that they are more resistant than cows to milk fat depression after fat addition to the diet (14, 15).

Goat milk is a great dietary source. It provides ample benefits for the health maintenance, physiological process, and in the nutrition of younger and the elderly population, and some studies have reported that it may be consumed by most of the population susceptible to cow milk allergy (16). Due to its non-allergic nature, digestibility, and contribution to the reduction of poverty and hunger at the community level in local producers and the ultimate consumer, the positioning of goat milk, should be on a global level (10). In terms of unique alkalinity, greater buffering capacity, and therapeutic potential in human nutrition and medicine, Goat milk varies from human milk or cow milk (3). Without the interventions of sanitary processes, and suitable manufacturing techniques specialized products derived from goat milk cannot be manufactured. However, issues about control of sanitation and the necessity to implement standards and quality tests remain a persistent problem for the marketing of goat milk and its products. A better incentive is payment based on the product quality, both in terms of microbiological condition (count of somatic cell and bacteria) and nutritional content (fat and protein) (17). It has long been recognized that goat milk naturally has a higher concentration of somatic cells than cow milk, owing to the mammary gland's apocrine secretory mechanism (18). A high somatic cell count (SCC) has a significant impact on the quality of goat milk. A study demonstrated that alterations in milk quality associated with SCC > 600.103/mL (5.58 LSCS) in a farm with extremely strict sanitation standards and no detectable mastitis-causing bacteria (MCB) in the mammary gland. The findings indicated that even a lower value of SSC can have a substantial effect on goat milk's technological features. The routine screening of SSC should be exhibited and reported to dairy manufacturers to ensure the consumer receives a high-quality end product (19).

The current trend toward the demand for healthy foods and in developed nations has developed in interest in goat milk and its products (1). Goat milk contains in abundance protein, fatty acids, and minerals (20). The excellent digestibility and nutritional content of goat milk, as well as its therapeutic potential, make it an important functional food (7). These characteristics pave way for the manufacturing of a wide range of dairy products from goat milk, including yogurt, cheese, non-fermented and fermented beverages, condensed milk, butter, ice cream, and sweets (21). This review generally focuses on the nutritional profile of goat milk, different preservation techniques in enhancing the quality and storage of goat milk, application of different processing technologies in the manufacturing of a wide range of goat milk-based products, and the potential health benefits of goat milk in human health and nutrition.

## Comparison of Goat Milk

### Comparison of Goat's Milk-Based on Pasture-Based and Indoor-Based Feeding System

Consumption of grass at the initial stage of growth positively influences the production of goat milk and its fat content. The modification of milk quality and its composition is largely dependent upon the variety of fodder species, stage of vegetation, stocking rate, and season. Pasture-based natural farming techniques generate milk that is high in fat and micronutrients (fatty acids, vitamins), as well as volatile components (flavor, terpenes). The comparison of three systems of feeding derived from natural pasture on the mountains, hills, and in the plains suggests that on the mountain pasture, the milk yield is rather lower but the content of protein, fat, and PUFA percentage are relatively higher (Table 1). The higher content of conjugated linoleic acid in milk is observed when the feed of grass obtained from natural pasture is given at an early stage. In intensive types of indoor systems, a high intake of fodders with high nutritional value or a plentiful supply of concentrates enables the production of milk that is high in protein and low in fat. The feeding of a lower-fat or fiber diet to goats can help in reversing the fat to protein ratio percentage, especially during mid-lactation. As a result, the lowering of cheese quality in terms of good taste and granular paste is observed. The increase of concentrate supply to 60% of total intake of dry matter in diet can result in a slow and linear decline of fat content, but when the intake concentration approaches 60–80%, there is a rapid decline in the fat content due to greater shortage of fibrosity in the ration (24).

**Table 1:** Comparison of fatty acid composition of goat milk based on pasture-based and indoor based feeding system.

**Fatty acid**	**Feeding based system**			
	**Renna et al. (22)**		**Pajor et al. (23)**	
	**Indoor-Based (Hay) (mg 100 g^−1^ dry matter)**	**Pastured-Based (mg 100 g^−1^ dry matter)**	**Indoor-based (Hay and concentrate) (mean ± SD), % of total FA**	**Pastured-based (mean ± SD), % of total FA**
α-linolenic acid	354.6	1,507.3 ± 395.34	0.71 ± 0.08	0.71 ± 0.13
Linoleic acid	188.4	344.0 ± 65.49	2.49 ± 0.15	1.98 ± 0.27
Vaccenic acid	2.9	6.2 ± 1.37	1.38 ± 0.23	1.69 ± 0.34
Saturated fatty acids	446.8	445.7 ± 32.72	69.12 ± 1.91	70.85 ± 3.59
Monounsaturated fatty acids	109.8	112.5 ± 8.56	26.85 ± 1.80	25.16 ± 3.28
Polyunsaturated fatty acids	543.1	1,857.4 ± 459.35	4.04 ± 0.19	4.00 ± 0.50
Total fatty acids	1,099.7	2,415.6 ± 492.04	–	–

The proportion of diet concentrate has a relative effect on the amount of iso-C15:0, iso-C16:0, and C13:0 in goat fat milk. Comparing conventional and organic farming systems with grazing and a smaller fraction of conserved forage often reveals a greater proportion of nutritionally beneficial fatty acids such as PUFA n3, branched fatty acids, and rumenic acid (25). In a study, the diet of Alpine goats was supplemented with hempseed or linseed, and later, the milk was analyzed after 14 weeks of treatment. The results demonstrated that feeding a seed enriched diet positively influenced the composition of fatty acids with an elevation in unsaturated fatty acids. Linolenic acid content was significantly increased in the linseed diet compared to the hempseed and the control diet. However, linoleic acid content was significantly elevated solely with hempseed supplementation. A variety of conjugated linoleic acid (CLA) isomers and greater levels of trans-configuration fatty acids were identified in supplemented diets, notably in the linseed diet (26).

### Comparison of Goat Milk With Cow Milk

Triglycerides are the primary components of milk fat, accounting for 97–98% of total milk lipids. The triglycerides composition of bovine milk fat is extremely complicated, containing around 1,300 distinct potential triglycerides if only the triglycerides whose content is above 1% are taken into account. Numerous studies indicate that dependence of availability of grass (which fluctuates seasonally), and supplementation with seeds such as soy meal, canola, or rapeseed can significantly enhance the CLA concentration of milk up to 50 percent. As a result, significant differences in the CLA content of goat and cow milk from different nations are to be expected (27, 28). There is extensive evidence indicating that the fat composition of goat and cow milk is significantly different. As a result, the composition of lipids from products derived from these milk types, such as cheese, will vary (29–32). The difference in triglycerides content of goat and cow milk is given in Table 2.

**Table 2:** Comparison of different triglycerides content of goat milk and cow milk.

	**Vieitez et al. (27)**		**Gastaldi et al. (28)**	
	**Goat milk (wt%)**	**Cow milk (wt%)**	**Goat milk [mol% (mean value ± SD)]**	**Cow milk [mol% (mean value ± SD)]**
C4:0	2.4	4.2	5.07 ± 0.023	8.45 ± 0.024
C6:0	2.8	2.6	3.78 ± 0.018	3.93 ± 0.018
C8:0	3.3	1.4	3.03 ± 0.029	2.08 ± 0.015
C10:0	11.5	2.8	17.2 ± 2.17	2.89 ± 0.38
C12:0	4.6	3.1	6.18 ± 0.093	3.21 ± 0.053
C14:0	10.0	11.2	12.9 ± 0.86	11.7 ± 0.78
C16:0	25.7	29.3	21.1 ± 0.33	21.9 ± 0.338
C18:0	9.7	9.9	8.11 ± 0.659	12.9 ± 1.06

## Processing of Goat Milk and Its Products

The fundamental processing techniques involved in the processing of fluid goat milk are similar to that of cow milk. Receiving milk, filtering, standardizing, pasteurizing, chilling, packing, storing, and distributing fluid goat milk are the key processes and techniques used. However, goat milk is commonly not required to be homogenized attributed to the prevalence of smaller fat globules than cow milk, resulting in a naturally homogenized consistency. After the milk is collected from an individual animal, a bulk tank, or a milk transportation truck, it is filtered to remove unwanted elements like sediments, udder body cells, and certain bacteria (33). Clarification is used in the removal of excess impurities through distributing the milk into thin layers over conical disks, revolving at a relatively high speed. Bactofuge, a unique machine that operates at considerably higher centrifugal force, has been developed for a high degree of bacterial decontamination (34). Depending upon the time and temperature relation, pasteurization can be performed as—Low-temperature long time (65°C, 30 min); high-temperature short time (72–75°C for 25 s); Ultra-high temperature (125 ± 5°C for 4s or 135 ± 5°C, 4s) (35–37). Pasteurization is critical to avoiding human brucellosis (multiple organ disease), which is frequently transmitted via unpasteurized goat milk and its cheese (38).

Following pasteurization, and chilling of the milk, it is bottled or packaged in paper or plastic cartons or other forms of packaging. This processed and packaged fluid milk is subsequently distributed to retail and other marketing outlets in refrigerated vans. The process of separation of cream in goat milk is optional, as goat milk cream is rarely found in commercial markets (33). The primary objective of cream separation is to standardize milk fat and produce butter, ice cream, and other dairy products. The cream is produced by the process of separation of skim milk from whole milk, resulting in the formation of fat-in-water emulsion enhanced with milk fat that has been heat-treated according to industry standards (39).

### Processing of Goat Milk Cheese

The first step in cheese making is obtaining high quality goat milk that is free from visible impurities, abnormal odor or taste, foreign substances, pathogenic microorganisms, and should possess desirable acidity (pH 6.2–6.55). Also, the cheese starter culture bacteria must survive and multiply after being added to the milk (40). The typical farmhouse goat milk cheese making process consists of the nine fundamental stages listed below (41): milk filtering → renneting → milk coagulation → putting the curds into appropriate cheese molds → draining → unmoulding → salting → drying → ripening (42). Although the core cheese making methods are identical in many goat cheese-producing nations, several distinct kinds of caprine cheeses may be created due to variations in the milk content, changes in the production procedures, and a wide range of maturing time and circumstances. The major types of cheese are (33, 43, 44):

#### Soft Goat Milk Cheese

Several thermophilic and mesophilic microorganisms or their combination, that is, *Lactococcus Lactis subsp. Lactis, Lactococcus Lactis subsp. cremoris*, and *Streptococcus thermophilus* as well as mesophilic ones including *Lactococcus Lactis subsp. lactis* and *Lactococcus Lactis subsp. cremoris* are commonly utilized starter cultures for soft goat milk cheese (45). Goat milk is pasteurized for 120 min at 62.8°C, followed by gradual coagulation and natural draining, and then wrapping in cheesecloth, subsequent hanging for 3 days in a cold environment (22°C) before packing. Polyolefin shrink wrap is used for the packaging of this type of cheese.

#### Semi-hard and Hard Cheese

The pasteurization of goat milk is exhibited at 62.8°C for 30 min. IFPL starter culture [contains the adjunct *Lactobacillus casei subsp. casei* (IFPL731) and isolated *Lactococcus lactis subsp. lactis (IFPL359), Lactobacillus plantarum* (IFPL935), *Leuconostoc paramesenteroides* (IFPL705), and *Leuconostoc mesenteroides subsp. dextranicum* (IFPL709)], homofermentative starter culture (EZAL^®^MA011, which contains *Lactococcus lactis subsp. cremoris* and *Lactococcus lactis* subsp. lactis) (46), *Lactococcus lactis subsp. lactis LPS31 strains, Lactobacillus casei subsp. casei 3PS103*, and *Streptococcus thermophilus* SPS1 (47), etc., are different starter cultures usually utilized for semi-hard and hard cheese. In addition to starting culture, single strength rennet in milk and then allowing it to coagulate. Cutting of curd and allowing it to heal for 5 min. The temperature is progressively increased to 39°C over 30 min and then cooking of curds until it becomes firm for 45–60 min. After draining two-thirds of the whey (31°C) warm water is poured into the vat for washing of curd and to bring the whey temperature at (31°C). Before the whey is entirely drained, the curds are steeped in water for 5 min. The curds are then pressed in vertical cheese press at 40 psi overnight for room temperature. The cheese is then taken from the molds, sliced into discs, vacuum-packed in plastic pouches using a vacuum packaging, and then stored at 4°C.

#### Blue-Veined Cheese

Roquefort and Savoy generally manufactured in France and Cabrale found in Spain are some of the popular Blue-veined cheese. The blue cheese curd is made with rennet and lactic acid culture. After curdling for 1–2 h and inoculation with penicillium, it displays the appearance of a bluish or greenish marble. After piercing and salting, the ripening of cheese is exhibited for a period of 1–4 months at a temperature of 9–10°C and relative humidity of 90–95% (48).

#### Acid-Coagulated Cheese

Crottin de Chavignol PDO, like the majority of cheese produced from French goat raw milk, is an acid-coagulated type of cheese. Milk is usually procured from Alpine goats. The culturing of commercial starters should be done from the delimited area of goat milk before being inoculated in vat milk. Frozen or fresh whey can also be utilized as a source of starter culture from previous cheesemaking and a minimum of two starter strains must be inoculated. After the process of pre-draining, frozen curd can be combined with fresh curd to achieve a ratio of frozen to fresh curd of 1:1. The time for transportation of cheese to the maturation plant and storing it before salting should not exceed 72 h, and it should be kept at a temperature lower than 10°C. The minimum period required for the maturation of cheese is 10 days to a maximum of 15 weeks. After the usual maturation, Chavignol “repasses” must be matured in the absence of airflow (49).

Apart from basic cheese manufacturing procedures, advanced techniques such as ultrafiltration can be utilized in enhancing the quality and yield of goat milk cheese. For the manufacture of retentate, considered as a fraction of pre-cheese from goat milk, ultrafiltration was utilized. The portion was then processed into cheese. Due to the maintenance of whey proteins with the curd and utilization of lower rennet, the rate of cheese production increased by 8 to 15% (50, 51). It is now feasible to make goat milk cheese with ultrafiltration technology even when there is a lack of fluid milk supply. The ultrafiltration method also offers benefits for the manufacturing of a cream cheese-type product, such that the pre-cheese material can be held for relatively more time in frozen storage for future use (50). The major obstacles to the manufacturing of goat milk cheese are a lack of αS1-casein, high somatic cell numbers, rancid flavor, and a greater concentration of free fatty acids (52).

### Processing of Goat Milk Yogurt

Goat milk yogurt products based on production technology are classified as below (33, 53):

(a) Set-type yogurt consisting of a firm gel that is the result of incubating milk with a starting culture in the final packaging.
(b) Stirred-type yogurt, having a relatively decreased firm texture than set-type yogurt. In this example, the coagulum is broken, agitated, and packed while incubating in a tank.
(c) Yogurt that has been strained or concentrated to have a creamier texture and a less acidic flavor. It can be made from stirred yogurt that has been concentrated by utilizing a cloth bag, ultrafiltration, or mechanical separation.
(d) Yogurt involving total solids of around ~26/100 g, comparable to a typical product, or made from recombined dairy components such as various milk powder and anhydrous milk fat, salt (optional), and stabilizer.

In an experiment performed by Jia et al. (54), the following process was utilized for the preparation of goat milk yogurt by using *Lactobacillus rhamnosus GG*. Firstly, skim milk sterilization was performed at 105°C [solids concentration of 11% (wt/wt)] for activation of strain. *L. delbrueckii ssp. bulgaricus, L. rhamnosus GG*, and *S. thermophilus* lyophilized powders were activated in skim milk and subsequently kept at 4°C. Sterilized Skim milk containing 5% inoculum of activated strain was used for the preparation of mother culture. The incubation of mother cultures of *L. delbrueckii ssp. bulgaricus* and *S. thermophilus* were conducted at 42°C and the incubation of *L. rhamnosus GG* mother culture at 37°C. They were then placed at a temperature of 4°C until the texture of the curd became firmer. A filter cloth was used to purify the raw goat milk. After the addition of sugar, pasteurization of goat milk was carried out at 95°C for 5 min and then subsequently cooled to 45°C. The goat milk was then inoculated with given strains in different ratios. Finally, the fermentation of goat milk was carried out at 4°C. Results suggested that high quality goat milk yogurt was obtained in addition to *Lactobacillus delbrueckii ssp. bulgaricus, Streptococcus thermophilus*, and *L. rhamnosus GG* in the ratio 1:1:3 at fermentation temperature of 42°C (54).

Sumarmono et al. (55) prepared stirred-type goat milk yogurt by using different cultures. Firstly, the pasteurization of milk was carried out by using low temperature long time methods at a temperature of 63°C for 30 min. Then the 5% (w/w) previously activated yogurt culture (lyophilized) containing *L. achidophylus, L. bulgaricus*, and *S. thermophiles* are added into stirred-type yogurt sample. At a temperature of 40°C for 5 h, the sample was incubated in a tightly sealed stainless-steel container. The yogurt was then moved into a sterile cheese cloth for partially removing its whey by tying up and hanging it in a cold room for 24 h. The end product was concentrated yogurt with a creamy texture and ~25 g 100 g^−1^ of total solid content (55).

Feng et al. (56) prepared a novel goat milk dairy product through the utilization of jujube pulp. Initially, raw goat milk (containing 3% fat and around 10% non-fat solids) was filtered, pasteurized, and then cooled to 4° C. Following that, concentrated jujube pulp was mixed with the goat milk at the concentrations (wt/wt) of 0, 3, 6, and 9% naming it as Y0, YJ3, YJ6, and YJ9 sample. The mixture was stirred continuously by placing it in a water bath at a temperature of 90 ± 2°C for 5 min. The samples were inoculated with 3% reactivated YO-MIX187 Yogurt cultures including *Lactobacillus delbrueckii ssp. bulgaricus* and *Streptococcus salivarius ssp. thermophilus* after cooling to 42°C. The inoculated samples were kept at 42 ± 2°C for 6 h until coagulation occurred, and then the resultant goat milk yogurt products were refrigerated at 4°C for 28 days. Triplicates of all jujube goat milk yogurt products were developed. YJ3 sample, on the other hand, has the desired water holding capacity, adhesiveness, and hardness. Additionally, the inclusion of jujube pulp significantly diminished the goaty flavor, enhancing the sensory acceptability, and increasing the antioxidant activity of goat milk yogurt (56).

### Processing of Evaporated Goat Milk Powder

The basic concept involved in the system of evaporation is the condensation of steam or vapor toward one end of a metal surface in heat exchangers, forcing the liquid on another end to develop into vapor. Evaporated goat milk is produced in the same manner as the products from cow milk. Evaporation is often performed at lower pressure for two reasons: first, to allow boiling at a relatively reduced temperature and therefore avoid heat damage, and second, to promote evaporation in more than one stage. The falling film type of evaporator is generally utilized in nearly all evaporation units. In this design, The flow of liquid is on the inner surface of several plates or pipes as a thin film through the action of gravity, while the presence of the heating medium is outside (57–60). For dried milk products, two distinct processing processes are utilized in the production of powdered milk: spray drying and roller drying. The process of spray drying converts fluid milk into a dried particle through the action of milk spraying into a hot drying medium. Conventional spray drying generally consists of four process stages: (i) milk atomization into a spray, (ii) spray drying through the contact of air (flow and mixing), (iii) spray drying through evaporation of water, and (iv) isolation of the dried product from the air (33). In the process of roller drying, processed milk or milk concentrate is spread as a thin film on the surface of the metal drum, which is rotating and steam-heated. The film of milk is dried and is continually scraped off by utilizing a stationary knife placed against the application point of concentrate during the rotating process (61).

Nascimento et al. (59) obtained goat's milk powder through the utilization of a semi-industrial spray dryer (LABMAQ model SD10) and open cycle mode in which atmospheric air acts as a drying gas. The goat milk used had a 13% (kg/kg) initial concentration of solids. The goat milk feed rate of 2 kg/h, the inlet temperature of 130 ± 2°C and outlet temperature of 90 ± 1°C of spray dryer, and 288 ± 5 kg/h of drying air-flow rate was used during the operation. A two-fluid nozzle with a 2 mm diameter of orifice and gas flow rate of 2 ± 0.1 kg/hr was utilized for atomization to produce droplets of goat milk. Since the monolayer value of 2.404% showed that the powder is better stable at a moisture content of 2.4%, it concluded that 2.4% should be a targeted moisture content for the powder after the spray drying process. According to the range of water activities when this moisture content is between 0.2 and 0.3, this conclusion is validated and is regarded as a safe storage zone (59). It is observed that *S. aureus* has the potential of spreading throughout the goat milk powder in different producing locations. Goat milk powder production stages are also prone to cross-contamination with *S. aureus*. The presence of *S. aureus* strains in the goat milk powder may pose a public health risk. Hence, proper care and safety protocols should be followed to avoid the possible contamination of *S. aureus* in goat milk powder (62).

## Preservation of Goat Milk by Novel Techniques

The goat milk business is unable to compete with its dairy cow counterpart in terms of the overall volume of output due to the lesser scale of milk production than for cow milk and the seasonal milk availability. This species-specific intrinsic disadvantage of the dairy goat business demands the investigation of various alternative and sophisticated technical solutions for year-round supplying of goat milk products, as well as improving productivity, quality, distribution, and storage (63). Cleaning udders before milking is required, with specific attention paid to end teats, employing predipping solutions, followed by utilization of paper towel to dry teats, resulting in avoidance of residues in milk. The person responsible for milking must be in good health, fully wash his or her hands, be smoke-free, and wear clean clothes. The initial squirt of milk must be collected in a black bottom jar when milking, as well as monitoring anomalies in milk and rejecting milk with a relatively greater bacterial count, to maintain higher milk quality. It is preferable to do CMTs (California Mastitis Tests). To obtain all of the milk, the milking process should be quick and calm. After milking, it is critical to undergo a dipping process in which the teats are immersed in a solution of glycerinated iodine and the goats are not allowed to lie down for a period of 30–60 min. When they return to the barn after milking, a smart strategy is to offer them the required diet (64).

Finally, there is the process of milk storage. When the milk initially exits the doe, it is usually at body temperature and therefore should be chilled to 4°C as quickly as possible for the optimum flavor and quality. Some manufacturers chill their milk in a freezer, ice, or cold water bath (63, 64). The cooling of goat milk must be exhibited immediately after milking to the 36–42°F holding a range of temperature, and it is mandatory in maintaining that temperature until the process of processing as well as throughout transit to a dairy plant (44).

### Preservation by Heat Treatment and low Temperature

Microbial growth has a detrimental effect on the physicochemical characteristics and shelf life of raw as well as processed milk (65, 66). The growth of microbes reduces the shelf life of pasteurized milk by generating unwanted characteristics and causing a change in sensory properties. As a result, customers have faced severe issues with microbiological safety and product quality preservation. Heat processing is an old, efficient, and easy method for treating dairy and its products. It is used to decrease the bacteria count in raw milk as well as to improve the sensory qualities of milk components and nutritional benefits (35, 67). The pasteurization process can help in the reduction of the bacterial count in raw milk, which is crucial for enhancing milk shelf life. The procedure, on the other hand, does not affect milk composition or fatty acid profile (68). Caprine milk's high ionic calcium concentration and poor micellular solvation may lead to thermal instability. Goat milk is considered to be extremely sensitive to heat treatment; it is typically unable to survive ultra-high temperature (UHT) treatment. Several techniques, including pH modification, the inclusion of a calcium sequestrant, and preheating of milk, have been believed to enhance heat stability and maintain UHT procedures for goat milk (37, 69). To avoid an enzymatic breakdown that may occur during the storage of goat milk, pasteurization can be performed before UHT sterilization. The indirect procedure is the most commonly used UHT treatment, although the direct process, which uses steam injection and infusion, can also be used. Heat stability of milk is critical in the production and processing of dairy products, particularly in the creation of goat milk products. The majority of heat stability tests on goat milk have revealed that it is quite sensitive to heat treatment (1, 33).

The research was carried out on the pasteurization of goat milk at different time and temperature combinations (72, 75, and 81°C for 15 and 25 s). The study found that heat treatments between 72°C for 25 s and 75°C for 25 s resulted in the greatest stability of pasteurized goat milk for up to the storage of 3 weeks at a temperature of 4°C. The two treatments had no significant impact on the nutritional, physicochemical, microbiological, antioxidant, and quality parameters of pasteurized goat milk. Although 81°C had a superior preservation effect, other goat milk-keeping characteristics were considerably impacted. As a consequence of the findings, suitable heat treatments can improve goat milk quality while decreasing psychrotrophic and mesophilic counts and extending goat milk's microbiological stability (70). It was demonstrated that the pasteurization technique of high-temperature short-time (HTST) was considered superior for preserving different vitamins and extending the shelf-life of goat milk. In terms of riboflavin, thiamine, and vitamin C preservation in goat milk, the flash, HTST, and UHT procedures outperformed the autoclave treatment and LTLT (low-temperature long-time) techniques. Pasteurization is carried out per standards of US FDA or EU requirements. The manual and batch type of milk pasteurization is generally done at 145°F (62.8°C) for 30 min (33, 71, 72).

The storage of raw goat milk at a temperature of 4°C for the period of 1, 3, and 5 days results in unaffected goat milk composition of lactose, ash, moisture, total solids, proteins, and fats (73). Similar results regarding the unaffected composition of goat milk during cold storage were observed in many other studies around the world. A study on the evaluation of refrigerated raw goat milk storage influencing the quality of whole milk powder suggested that to protect the quality of goat milk powder during its 180-day shelf life, raw goat milk should be stored at 4°C for no more than 3 days (73). The time-temperature indicator, also known as the time-temperature integrator (TTI), is a straightforward smart packaging indication that uses natural dyes as a source of indicators. The dye may vary in reaction to product changes during storage. The utilization of synthetic dyes such as bromothymol blue and methyl red or natural types of dyes involving yellow betaxanthin pigments and red-violet betacyanin, obtained from red dragon fruit can be exhibited (74, 75), conducted a study to assess the fresh quality of goat milk when stored for 31 days at freezing temperatures (−20 ± 2°C) and 24 h at room temperature (25 ± 2°C), by utilization of bioindicator films placed externally on milk bottles. The results revealed that color alterations in the bioindicator film were more visible at room temperature, compared to that of freezing temperature. Based on the color variations of the bioindicator at room temperature, the consumption of the sample was considered safe until the fifth hour with a pH of 6.51. Fresh goat milk with a pH of 6.51 was safe to consume even at freezing conditions until the 31st day.

### Hydrostatic High-Pressure Treatment

The treatment by HHP is a novel method of food preservation. This procedure can improve texture as well as the presence of dormant harmful bacteria (76). It has been utilized in the enhancement of goat milk and increased the retention of nutritional content, color, and flavor (77, 78). HHP may also affect rates of an enzymatic reaction, and its usage may result in denaturation of whey proteins, disrupting casein micelles, and changing the textural properties (79). High pressure processing entails semicontinuous or batch treatment of product around 800 MPa (8,000 bar) pressure. The flexible packaging is generally utilized for the packaging of the product, consisting of little headspace and usually placed in a vessel filled with water or containing different pressure-transmitting fluid. After that, the vessel is sealed, and the fluid (responsible for transmitting the pressure) is injected into the system until the necessary pressure is attained. The system is retained at this pressure for the required holding period, and then the pressure is released, allowing the vessel to be opened, the treated product removed, and the new product fed into the system for the next cycle (80). The degree of microbial inactivation is determined by the pressure used, the temperature, the environment, the duration of treatment, and the quantity and kind of microorganisms. In terms of technology, HHP provides a possible pasteurization alternative to heat treatment. The application of pressures between 300 and 600 MPa inactivate molds, yeasts, and the majority of vegetative bacteria. Only bacterial spores can withstand pressures beyond 1,000 MPa pressure, although spores can be inactivated by heat treatment or moderate pressure if germinated at 50–300 MPa (81).

### Ultrasound Treatment

In comparison to other processing methods, the utilization of ultrasound techniques in food processing is pretty new (82). It is considered to be a non-toxic and environmentally friendly method used in the North American dairy industry to homogenize and emulsify milk. It enhances the texture and viscosity properties of fermented products through the application of high power (above 100 W) and a low frequency of around 20 and 45 kHz. It may also aid in enhancing the products' soluble phosphorus and calcium content, which is directly connected to the textural and rheological features of fermented dairy products (76). When employed at high frequencies (18–100 kHz), ultrasound can stimulate or kill live cells, alter enzyme activities, increase extraction or impregnation of components, and enhance emulsion, freezing, and crystallization processes (82). The technique used in ultrasound is based on acoustic waves flowing through a specified material at a relative speed determined by the type of the wave and the substance through which it travels. Bacteria can be inactivated by ultrasound therapy, however, the amount of inactivation is dependent on the kind of bacterial cell and various treatment circumstances (80).

The major phenomenon associated with the ultrasonic process is “cavitation,” in which through the action of rapid collapse and rectified diffusion, liquid bubbles are developed. The implosion and oscillation of bubble cavitation can lead to disruption of cells, shock waves, shear forces, change in pressure and temperature, production of free radicals as well as turbulence (83). Ragab et al. treated goat milk with ultrasound for 15–30 min at a frequency and power output of 20 kHz and 12.1 ± 0.89 W, respectively. The results showed that the shear forces produced from ultrasound decreased particle size, changed the protein secondary structure, and enhanced the content of its β-sheet, resulting in improving its surface hydrophobicity. The prolonged period of ultrasonic treatment resulted in the enhancement of microstructure, elasticity, and rheological property of the rennet-induced gel. As a result, ultrasonic technology might be used in the dairy industry to improve the quality of rennet coagulation during the manufacturing of goat milk cheese (83). In addition, after being exposed to ultrasound for 6 min, the apparent viscosity and consistency index of several yogurt samples obtained from goat milk was considerably improved (84).

### Ultrafiltration Treatment

The advanced separation technologies serve as the foundation for adding value to milk processing (85). The dairy industry utilizes the technique of membrane separation such as reverse osmosis and ultrafiltration in concentrating and removing components from milk. These procedures are also utilized to enhance the dry matter of milk, which benefits dairy products' textural and rheological characteristics (76, 86). Membranes are already used in a variety of dairy technology sectors such as processing of whey, fractionation of milk fat, processing of milk protein, and demineralization or desalination (87).

### Microfiltration Treatment

Microfiltration is a type of membrane separation technique identical to ultrafiltration except that the membrane pore sizes are larger, allowing particles with diameters ranging from 0.2 to 2 μm to pass through it. It is a non-thermal type of treatment method for reducing the presence of bacteria and improving the microbiological safety of dairy products while retaining flavor and taste (87). Due to the capability of removal of cells, microfiltration can also be utilized by the dairy sector to lower somatic cell count. However, because of size changes in fat particles, this treatment may alter the technical milk appropriateness for the processing of other products (77). Microfiltration seems to be a promising approach for producing whey fruit juice beverages with similar rheological characteristics used in the dairy sector to make whey fruit juice beverages with comparable rheological properties to samples that are conventionally processed at lower temperatures, that could be beneficial for nutritional compounds (88). Vieira et al. evaluated the functional, microbiological, and physicochemical qualities of a novel microfiltered goat whey orange juice beverage (GWOB). When compared to the standard LTLT treatment (63°C/30 min), the different temperatures of feed at 20, 30, 40, and 50°C impacted the microfiltration (0.2 μm) of the GWOBs. The temperature of microfiltration affected the rheological properties and adopting 20° and 30°C temperatures kept a similar consistency to that of the LTLT sample while maintaining the texture components. Thus, it is suggested that GWOB should be processed through microfiltration at optimum temperatures of 30°-40°C to preserve homogeneity, volatile components, functional properties and obtain better quality of microbial population (88).

### Pulse Electric Field Treatment

The principle of food products treated with PEF is to focus on the development of high-intensity electric fields to products in the form of relatively shorter pulses. Field intensities are typically in the 10–80 kV/cm range, with pulse durations ranging from 0.1 to 5 μs. The design of the treatment chamber should allow for the establishment of a spatially uniform field distribution, the minimization of dwell time dispersion, and the regulation of temperature. PEF may be used in a wide range of waveforms. Exponentially decaying pulses, which consist of a unidirectional voltage that gradually rises to a peak and then exponentially decays to zero, are commonly employed. However, square and bipolar types of waveform pulses are more effective than exponentially declining waveform pulses for the inactivation of microbes (80).

PEF is a new alternative pasteurizing process that, when used appropriately, has the potential to increase the shelf life while reducing the negative effects on the organoleptic characteristics of goat milk. The findings indicate that monopolar PEF pasteurization can be suggested to improve the goat milk quality at the industrial level. Because of the growing need for greater nutritional content and fresh-like characteristics in processed foods, non-thermal treatments such as PEF have gained traction as a replacement for thermal pasteurization (57, 89–91). PEF is used at room temperature (20–25°C) and has a short treatment period, generally <1 s. Because of its cheap processing cost, little energy loss, and environmental friendliness, the outcomes of PEF processing are encouraging in the food and beverage production industries (57, 89). The research on the application of PEF on goat milk suggested that treatment of PEF at 40 kV/cm for 10 s resulted in substantial alterations in the titratable acidity and pH value of the treated samples due to a reduction in calcium ion (Ca2^+^), which leads to the phosphate protonation. At 20 kV/cm for period of 5 s, other physicochemical properties such as color, TSS, and viscosity were shown to have non-significant impacts. Furthermore, it was discovered that the PEF processing and parameters utilized in this study were free from contamination of heavy metal. To summarize, this study found that PEF has a high potential for utilization in treatment of milk pasteurization due to little changes in the organoleptic and physicochemical features of treated goat milk during milk processing (92).

## Health Benefits

**Figure 1:**
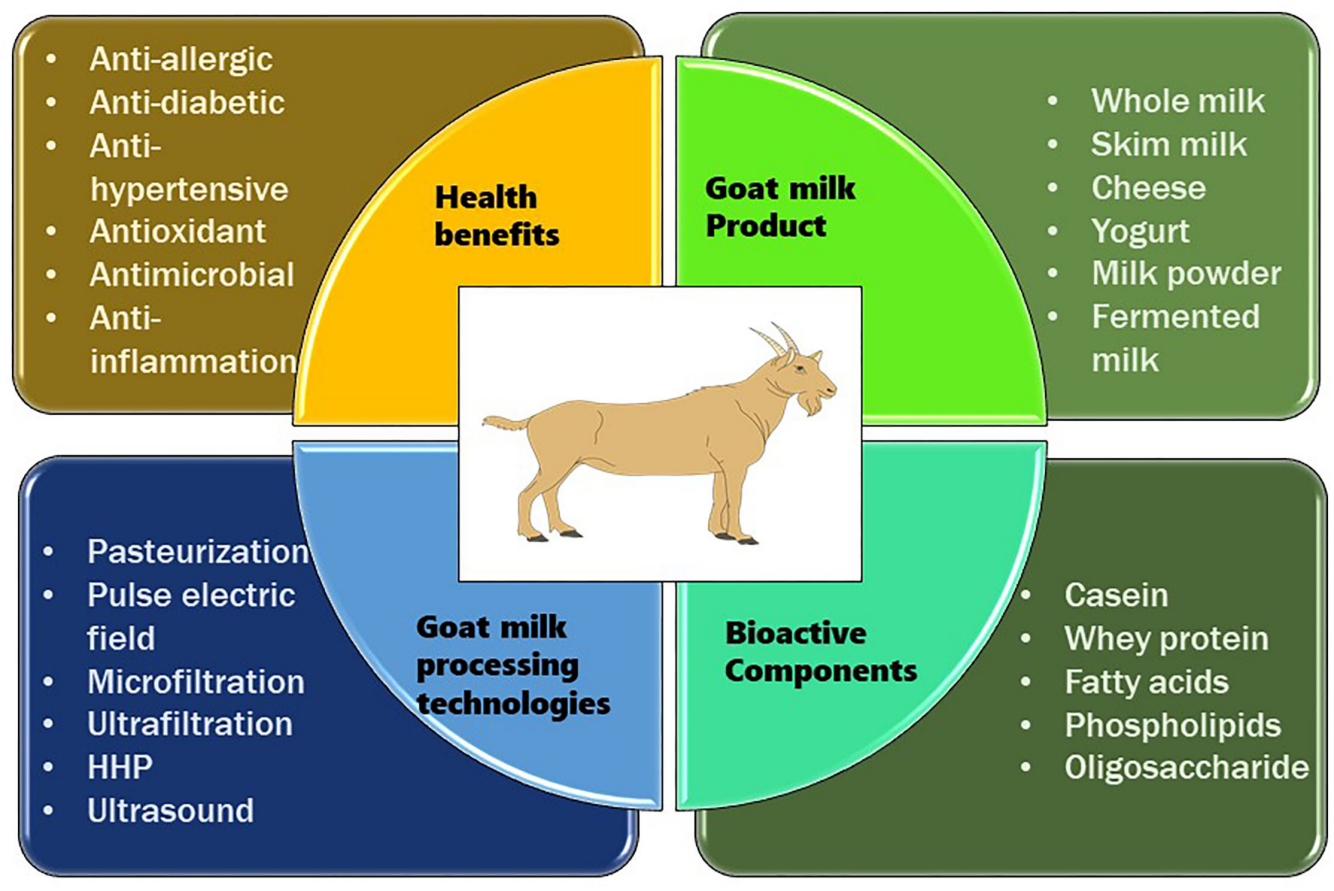
Processing, bioactive compounds, & health benefits of goat milk.

Milk is a source of nutrition for newborn mammals, as well as children and adults, and it is used for growth and sustenance. It is frequently regarded as a functional food because it exhibits potential bioactive compounds, such as whey proteins and casein, which are discovered to be highly significant for biochemical and physiological functions with a profound influence on human health and metabolism (10). In the current scenario, there is an increase in the importance of goat milk due to its unique composition and thus it is utilized as a superior raw material for the production of food for infants and the elderly, as well as for some segments of the population with special dietary requirements (93, 94). Because of its nutritional richness, digestibility, dietary and therapeutic potential, goat milk is becoming an indispensable part of the human diet (3). In addition to the presence of high-quality protein, goat milk comprises unsaturated fatty acids, vitamins, hormones, cytokines, enzymes, growth factors, and bioactive peptides, all of which assist to nourish and protect infants (95–97). Besides nutritional benefits of milk components, additional components such as antibodies, glycoproteins, and oligosaccharides can protect infants by preventing pathogen infections and supporting the growth of the intestinal epithelium (98). Various health benefits of goat milk are shown in Table 3. A general overview of the processing of goat milk, bioactive compounds, and its health benefits are shown in Figure 1.

**Table 3:** Health benefits of goat milk.

**Sr. No**.	**Milk component**	**Health benefit**	**The possible outcome from the study/research**	**References**
1	Casein	Anti-viral property	According to preliminary data, the process is non-specific and is mediated by a casein fraction component. This fraction is most likely comprised of one or more distinct components that interact with the capsid receptors or membrane of different viruses, therefore preventing cell entry and replication of pseudo virus SARS-CoV-2, Coxsackievirus A9, and HSV-1 viruses	(99)
		Anti-diabetic	Casein hydrolysates present in goat milk have the potential to improvise insulin resistance and treatment of type-2 diabetes	(100)
		Anti-hypertensive and immuno-stimulating	Caprine milk β-Casein can generate immune-stimulating and anti-hypertensive peptides	(101)
		Angiotensin-converting enzyme inhibitors (ACE inhibitors)	Identification of β-Casein f58–65 and αs2-Casein f182–187 in caprine milk have the potential in producing ACE-inhibitory peptides	(1)
		Anticariogenic effects	Calcium-binding Casein phosphopeptides (CPP) present possess anticariogenic properties via preventing caries lesions by recalcification of the tooth enamel, as well as competing for calcium from dental plaque-forming bacteria	(1)
		Antioxidant property	Peptides produced from αs-casein exhibit free radical-scavenging action and prevent both non-enzymatic and enzymatic lipid peroxidation	(1)
		Antagonistic or agonistic activity	The peptides present in β- and α-casein are known to function as opioid peptides, exhibiting antagonistic, or agonistic effects	(102)
		Cytomodulatory effect	Caseinophosphopeptides (CPPs) have been shown to have cytomodulatory properties by suppressing the development of cancer cells or promote the function of immunocompetent cells	(103)
2	Casein and whey protein	Immunomodulatory effect	Immunomodulatory effects of peptides and protein hydrolysates obtained from major whey proteins and milk caseins include the proliferation of lymphocytes, production of antibodies, and cytokine modulation	(10)
3	Whey protein	Antioxidant property	Whey protein processed at low temperature includes a relatively increased number of certain dipeptides (glutamylcysteine), which can increase glutathione production, an essential antioxidant essential in cellular protection and repair activities	(104)
		Anti-appetizing effect	The total content of whey protein present in the diet has been related to decreased LDL cholesterol and increased production of cholecystokinin (appetite-suppressing hormone)	(10)
		ACE inhibitors	The hydrolysate of caprine (β-Lg) produced with termolisin yielded four novel ACE-inhibitory peptides	(1)
4	Milk protein	Anti-thrombotic activity	Caprine κ-CMP and its hydrolysates with trypsin inhibited the aggregation of human platelet	(105)
5	Lactoferrin	Anti-microbial activity	A peptide derived from lactoferrin (Lactoferricins) has anti-microbial action against a variety of Gram-negative and positive- bacteria, fungi, and yeast	(106, 107)
6	Fatty acids	Hypocholesterolaemia	Fatty acids present are known to exhibit a hypocholesterolemic effect on blood and tissue through inhibition of dissolution and deposition of cholesterol in gallstones	(1)
7	Phospholipid	–	Phospholipids aid in fat absorption by forming a barrier around the fat globules, which keeps them finely distributed. Through their lipotropic action, phospholipids aid in the transfer of fat from the liver	(1, 104)
8	Medium-chain triglyceride (fat)	Energy providing effect	Because MCT is a readily accessible energy substrate, goat milk has a substantial influence on supplying energy, particularly in developing youngsters	(108)
9	Cholesterol	–	It is considered a metabolic precursor of bile acid and vitamin D. It is essential for metabolic processes involved in DNA synthesis, transportation of lipid, and cell division	(102)
10	Oligosaccharide	Cell protection activity	They help in the protection of intestinal mucosa cells against infections by encouraging the development of *Lactobacillus bifidus* in the digestive tract, particularly in infants	(109)
		Anti-inflammation	They help in decreasing intestinal inflammation and aid in the repair of damaged colonic mucosa in rat studies	(110)
		Prebiotic anti-pathogenic effect	The oligosaccharides present in goat milk help in exhibiting anti-pathogenic and prebiotic effects, associated with enhancement of central nervous system and can be used in supplementation of milk formulation as an alternative to other milk	(111, 112)
11	Minerals	Enhanced mineral uptake	In rats, a goat milk-based diet boosted iron deposition in target organs while decreasing anemia	(113)
		Increase mineral bioavailability	The consumption of goat milk in rats resulted in increased bioavailability of selenium, zinc, and copper than cow milk-fed rats	(114, 115)
12	–	Increase in overall nutrition	Rats fed GM thrived substantially better, had greater liver weights, enhancement in hemoglobin iron content, and its absorption	(1)
13	–	Allergy	Goat milk delivery and feeding alleviated gastrointestinal allergies in some infants	(104)
14	–	Promotion of growth factors	GM contains a significantly greater concentration of growth factors, and a human epidermal growth factor (hEGF) polyclonal antibody was used to detect the existence of EGF in caprine milk	(18)
15	–	Antioxidant property	GM fermented with Lactobacillus fermentum (M4) had remarkable antioxidant activities	(116)

Goat milk has been known for its therapeutic and hypoallergenic characteristics in human health and nutrition, implying that caprine milk may include metabolically active and bioactive components that are specific to this species' milk. Considering the bioactive milk components, goat milk's hypoallergenic properties are crucial to human health and medicine. This concept has gained the interest of goat milk consumers and producers worldwide, particularly in developed nations in recent years (104). In a recent study, goat milk treatment cured a large population of children suffering from cow milk allergies; and in another allergy case study, 49 of 55 treated children benefited from goat milk treatment (102). Grant et al. observed a non-significant increased weight gain in infants (*N* = 36), which were fed with infant formula based on goat milk than in those fed with infant formula based on cow milk, even though the study of both formulae had identical macronutrient and nutritional (energy) composition (117).

Goat milk has a better buffering capacity than cow milk due to its relatively higher content of protein, phosphate content, and non-protein N. Some of the physicochemical characteristics of goat milk, such as shorter fat globules, softer curd creation, and a greater percentage of medium and short-chain fatty acids, are beneficial for higher digestion and better metabolism of lipid when compared to cow milk (10). In their study (118) found that giving goat milk rather than cow milk in the diet resulted in higher biliary production of cholesterol and a decrease in the content of plasma cholesterol. The bile acid and phospholipid outputs, as well as the lithogenic index, remained within normal limits. Furthermore, consumption of goat milk reduces plasma triglyceride concentrations and thus has a positive effect on the metabolism of lipid identical to that of consumption of diet including virgin olive oil (64). Several investigations have reported goat milk as a functional food attributed to its nutritional profile and health benefits, beneficial alteration of physiological processes, and decrease of risk from chronic ailments (3). Furthermore, it has been demonstrated that daily consumption of goat milk reduces acute inflammation, lowers exaggerated basal secretion of interleukin IL-6 (IL)-8 and acute response, and generates a modest down-regulation of IL-1β and production of tumor necrosis factor-α in immune-compromised elderly patients (48, 119). The ratio of omega-6 to omega-3 fatty acids in goat milk fat is 5:1, which is comparable to the ratio suggested for human cardiovascular disease prevention. Because CLA consumption can reduce body fat by blocking lipogenesis and increasing lypolysis, pasture-raised goats are the optimal strategy to enhance milk CLA content (120). In hypercholesterolaemic rats, goat milk casein and fat fraction lowered plasma cholesterol, elevated High-density lipoprotein cholesterol, enhanced fecal cholesterol excretion, and demonstrated antioxidative characteristics. These goat milk properties suggest that it may protect against disorders associated with higher cholesterol levels, such as atherosclerosis, as well as decrease the accumulation of free radicals and ROS, thereby protecting cell membranes from damage by oxidation (121).

Dairy goats are being used in livestock interventions in Malawi and Sub-Saharan Africa, where there are few or no infant formulas available for HIV-positive mothers in impoverished rural families. Mothers with HIV/AIDS are encouraged to exclusively breastfeed their newborns for the first 6 months since there is a decreased chance of viral transmission to the child during this time. After the period of 6 months, mothers are encouraged to discontinue breastfeeding and utilize milk replacements that are inexpensive, acceptable, readily available, sustainable, and safe. For this purpose, goat milk is considered to be an excellent choice (122). Furthermore, the inclusion of probiotics–live bacteria that, when consumed in sufficient quantities, provide advantages to the host–can increase the functional value of goat milk (21). Consumer demand has driven the dairy sector to create products with useful components, such as the inclusion of prebiotic compounds or probiotic microorganisms (123). The goat milk based functional products containing probiotics and prebiotics has received the dairy industry's attention for commercial and scientific reasons attributed to their immense health benefits (3). goat milk was reported to have a distinct antiviral impact, which is reduced but not eliminated by pasteurization. Goat milk outperformed bovine milk in terms of antiviral effectiveness. Similarly, goat milk aids in suppressing other viruses such as pseudovirus SARS-CoV-2 and Coxsackievirus A9 in addition to HSV-1 (99, 124). Thus, goat milk is more than just a “grandmother medicine”; this action is caused by casein in goat milk, and pasteurization decreases this effect (99).

Goat milk products are regarded to be the most marketable dairy products. As a result, various features of goat milk are generating significant research attention at the moment (48). The white brined goat milk cheese made with *Lactobacillus casei* and *Lactobacillus bulgaricus* possess the highest ACE-inhibitory and antioxidant activity (125). The antioxidant characteristics of goat milk kefir fermented with kefir grains were mostly determined by the fermentation and storage times, and the product demonstrated excellent stability in ABTS, FRAPS, and DPPH assays (126). Crude protein extract derived from goat milk whey has the potential to act as a bacteriostatic, cytotoxic compound for the destruction of tumor cells, and a potent antioxidant (127). The preventive effects of goat milk and goat yogurt on intestinal damage caused by acetic acid are similar to those of sulfasalazine, suggesting that goat milk and goat yogurt could be used as functional foods in inflammatory bowel disease (128).

The anti-inflammatory activities of lipids in goat and sheep products have been studied in traditional Greek Ladotyri and Kefalotyri sheep cheeses (129), and the lipid fractions of both kinds of cheese exhibited inhibitory activity toward PAF-induced platelet aggregation. Further analysis of the lipid fractions in both cheese, by thin layer chromatography, has shown that the most biologically active lipid fractions contained sphingomyelin, phosphatidylcholine, and phosphatidylethanolamine lipid derivatives. However, these lipid fractions do not always present as a typical phospholipid structure; they share a similar structure to phosphatidylcholine derivatives as reported by Nasopoulou et al. (130). Poutzalis et al. compared the PAF-inhibiting properties of goat products (milk, yogurt, and cheese), and all goat samples possessed PAF inhibitors (131). The presence of a greater concentration of medium- and short-chain triglycerides in goat milk have also been used to treat individuals with malabsorption who have chyluria, steatorrhea, intestinal resection, hyperlipoproteinemia, childhood epilepsy, coronary bypass, cystic fibrosis, premature infant feeding, and gallstones (3, 10, 48). goat milk is said to have a higher buffering capacity, which might be useful in stomach ulcers treatment. The utilization of whole goat milk as the sole protein source in infant formulas maximizes the level of fat, milk fat globule membrane, sn-2 palmitic acid, and medium and short chain fatty acids. These characteristics enhance the microstructure and composition of whole goat milk-based infant formula, emulating the complex fat globules seen in human milk fat, and have been demonstrated to aid digestion, as well as cognitive and immunological development (132). Goat milk, particularly in developing nations, is a viable dairy alternative for meeting the nutritional needs of children, infants, and adults (1).

## What Does the Current Review Add To the Existing Knowledge?

The previously published reviews, generally have focused on providing information on the nutritional content, processing technologies and health benefits of goat milk and its product (3, 10, 16, 18, 33, 48, 94, 105). This review provides the detailed comparison of the goat's feeding system in terms of pasture and indoor-based feeding, which conclusively suggested that pasture-based feeding enhances the fatty acid content of goat milk, resulting thus, in higher economic value and consumer benefit (24). The present review also highlights the recent insights about some alternative goat milk processing methods. The novel information about the production of different goat milk products incorporated with fruit juice, such as the preparation of goat milk yogurt with the inclusion of jujube pulp (56) and development of goat whey orange juice beverage using the microfiltration process (88), which not only masks the goaty flavor but also improves the overall nutritional and sensory profile of goat milk. More detailed research surely could set the basis for encouraging the development of functional beverages. Similarly, the utilization of bio-indicators described in the present study, developed for evaluating the quality and microbiology of goat milk at different times and temperature combinations could be considered as a novel method. This approach could be adopted for authenticating the quality of goat milk on commercial scale (75). Previous studies on goat milk suggeested the heat-dependent pasteurization methods, but the latest research proved that pulsed electric field treatment could be used as an alternative to the conventional pasteurization method to obtain better results. Goat milk treated with 20 kV/cm for a period of 5s showed similar results to conventional pasteurization, revealing its high potential for application as an alternative to conventional pasteurization method (92). Previous studies do not have definite information about the actual process and processing parameters for obtaining goat milk powder, but the study of Nascimento et al. (59) suggested some parameters in spray drying and other aspects of the process, to obtain an end product with very low moisture content (2.4%) and water activity (0.2–0.3), enhancing the shelf life and consumer acceptability of goat milk powder (59).

## Conclusion

Goat milk could be considered as a significant option for consumption in nearly all age groups, compared to other milk obtained from different animal species. Apart from imparting various nutritional benefits, goat milk promotes a wide range of health benefits in humans. Various technologies have been used to improve the textural, sensory, and overall quality of goat milk and its products, resulting in increased customer acceptance. Bioactive peptides present in goat milk-based products have tremendous therapeutic potential by regulating the physiological and metabolic functions of the body. Goat milk and milk-based products exhibit excellent cholesterol properties by improving cholesterol mobilization and controlling its storage in the blood. Serum protein hydrolysates of goat milk have been reported to have ACE inhibitory activity, particularly β-lactoglobulin. The amino acid sequence of the ACE inhibitory peptides found in goat milk may also form the basis for the formulation of products with significant therapeutic potential. Angiotensin I-converting enzyme (ACE) inhibitors present in goat milk products have shown potential antihypertensive properties. Functional and nutraceutical properties of goat milk could be attributed to its strong bioactive potential and hence could be considered for future applications in the development of functional foods for the treatment of certain chronic diseases. Exploiting the use of novel technologies in the processing of goat milk and its product could pave way for new horizons in the development of functional foods based on goat milk attributed to its significant bioactive potential.

Looking at the processing potential of goat milk, it can be utilized for the formulation of numerous common dairy ingredients such as ice cream, butter, etc. as well as for the development of novel dairy products. It can also be used to develop different infant-based foods, and will surely be a game-changer in infant foods if more and more detailed research is carried out. Goat milk is rich in bioactive compounds and functional ingredients, so its potential could be exploredfor the development of dairy based functional foods and nutraceuticals.

## Author Contributions

GN, MA, SA, and AHD contributed to the conception and design of the study. AHD proposed the title of the manuscript. YJ, SG, HH, and AND wrote the first draft of the manuscript. GN, DD, and OB wrote sections of the manuscript. MR, AHD, and GN have revised the manuscript critically. All authors collated papers, read, and approved the final version of the manuscript.

## Conflict of Interest

The authors declare that the research was conducted in the absence of any commercial or financial relationships that could be construed as a potential conflict of interest.

## Publisher's Note

All claims expressed in this article are solely those of the authors and do not necessarily represent those of their affiliated organizations, or those of the publisher, the editors and the reviewers. Any product that may be evaluated in this article, or claim that may be made by its manufacturer, is not guaranteed or endorsed by the publisher.
